# Reply to: Senescence, trait parameterization and (st)age-specific forces of selection

**DOI:** 10.1098/rspb.2021.2610

**Published:** 2022-03-30

**Authors:** Mark Roper, Roberto Salguero-Gómez

**Affiliations:** Department of Zoology, University of Oxford, UK

We respond to the Comment of Bahry [1] regarding our review of why and where selection gradients might not decline with age [2]). Bahry [1] posits that Roper *et al.* [2] ‘repeat a subtle mischaracterization of the relationship between Hamilton's indicators of selection and Caswell's generalized fitness sensitivity for (st)age-structured population projection matrices'.

However, we note that equations 3.1 and 3.2 of Roper *et al.* [2] are not the discrete time equations 9.1 and 9.2 of Bahry [1] and are instead equivalent to continuous time reformulations of Hamilton's seminal work [3] by Caswell ([4]—equations 1 and 2). Caswell [4] displays the equivalence of their Equations (1) and (2) and Hamilton's forces of selection in §2.2 of his work. We acknowledge that this confusion has arisen due to the reference of Caswell (1978) rather than Caswell (2010) in our original piece. We agree with Bahry [1]'s detailed comparison between Hamilton (1966) and Caswell (1978) and thank the author for creating the opportunity to provide further clarity. We would also like to clarify that Roper *et al.* [2] did not intend to assign equivalence between equations 6.1 and 6.2, and 9.1 and 9.2, of Bahry [1], even though, as the author notes, the differences between the continuous time and discrete time versions are trivial anyway.

To avoid any further confusion for the reader and the discipline, we have included table 1, which displays comparisons between the work of Hamilton (1966), Caswell (1978) and Caswell (2010). As displayed in table 1, and in the response by Bahry [1], Caswell (1978) generalized Hamilton's (1966) sensitivity analysis approach to stage-structured populations with discrete time, in which the rate of population growth *λ*, rather than the intrinsic rate of natural increase *r*, is the measure of fitness. This approach differs in assuming the genetic effects on survival are additive, rather than multiplicative (Hamilton (1966) assumed genetic effects subtract from mortality risk [1]), and that fecundity is ‘effective’ (i.e. it includes survival to the first age class) rather than indicating the total number of offspring produced. These assumptions lead to slight differences in the forces of selection acting on genes that affect mortality risk (or survival). In particular, it is residual reproductive value at age *x* in discrete time versus reproductive value in continuous time that balances the force of selection. For reproduction, if reproductive value at the first age class is defined as 1, then there are no differences between the discrete and continuous time forces of selection.

**Table 1.** Comparisons of the force of selection on mortality and fertility in Hamilton (1966), Caswell (1978) and Caswell (2010). The intrinsic natural rate of increase, *r*, is derived as the single real root of the Euler–Lotka equation in continuous time modelled populations, whereas the population growth rate *λ* (= *e^r^*) is the dominant eigenvalue of a population projection matrix that considers a population in discrete time. Caswell (1978) considered the sensitivity analysis approach in discrete time, which leads to slight differences in the forces of selection. On the other hand, Caswell's (2010) modifications showed that *l*(*x*)*e*^−^*^rx^* = *c*(*x*)/*b*, where *c*(*x*)is the stable age distribution of age class *x* and $b\;\left({\left[\int_{0}^{\infty }e^{-rx}l(x)\;dx\right]}^{-1}\right)$ is the birth rate. Equivalence between Hamitlon (1966) and Caswell (2010) can be demonstrated when these relationships are inputted into Hamilton's forces of selection (rows 4 and 6 of the first column). Other definitions from the table: *T* = generation time, *l*(*x*) = survival probability from birth to age *x*, *p_x_* = survival between age *x*-1 and *x*, *F_x_* = effective fecundity at age *x*, *m*(*x*) = fecundity at age *x* and *v*(*x*) = reproductive value at age *x*.

	Hamilton 1966	Caswell 1978	Caswell 2010
measure of fitness	*r*	*λ*	*r*
assumed form of genetic effect on survival probability	multiplicative	additive	multiplicative
assumed form of reproduction	age-specific fecundity (*m_x_*)	effective fecundity (*F_x_*) – includes survival to age 1	(st)age-specific fecundity (*m_x_*)
how is time modelled?	interval/continuous	discrete	interval/continuous
force of selection on reproduction	$\frac{\partial r}{\partial m(x)}=\;\frac{e^{-rx}l(x)}{T}$	$\frac{\partial \lambda }{\partial F_{x}}\;\propto \;c_{x}v_{1}$	$\frac{\partial r}{\partial m(x)}=\;\frac{c(x)}{bT}$
mathematical relationship to Hamilton 1966	N/A	$\frac{\partial r}{\partial m(x)}=\;\frac{\;p_{0}}{\lambda }\left(\frac{\partial \lambda }{\partial F_{x}}\right)=\frac{e^{-rx}l(x)}{T}$	*l*(*x*)*e*^−^*^rx^* = *c*(*x*)/*b*
force of selection on mortality/survival	$\frac{\partial r}{\partial \mu (x)}=-\;\frac{\int_{x}^{\infty }e^{-rx}l(y)m(y)dy}{T}$	$\frac{\partial \lambda }{\partial p_{x}}\;\propto \;c_{x}v_{x+1}$	$\frac{\partial r}{\partial \mu (x)}=\;\frac{-c(x)v(x)}{bT}$
mathematical relationship to Hamilton 1996	N/A	$-\frac{\partial r}{\partial \mu (x)}=\frac{\partial r}{\partial lnp_{x}}=\;\frac{\;p_{x}}{\lambda }\left(\frac{\partial \lambda }{\partial p_{x}}\right)=\frac{e^{-rx}l(x)RRV_{x}}{T}$	$\int_{x}^{\infty }e^{-rx}l(y)m(y)dy=l(x)e^{-rx}v(x)$

To summarize, the logic of Roper *et al.* [2] was to highlight the fundamental importance of the stable (st)age and reproductive value distributions when considering variation in the evolution of senescence. Equations 3.1 and 3.2 [2] were used as conceptual springboards, from which to (i) compare stable (st)age and reproductive value distributions against rates of senescence for species using matrix population models [5,6] and (ii) propose why and where across the tree of life one might expect a decline in selection pressure with age not to be as pronounced, contrary to predictions from Hamilton (1966) on the need for senescence to be universal. Bahry [1] is correct in noting that, depending on the assumptions one makes about the action of genes, selection gradients may differ in form [7]. However, this is a question of mathematical parameterization and does not detract from the main biologically driven argument of Roper *et al.* [2]: that the strength of selection on a (st)age class with a given life cycle will be proportional to the genetic contribution to future populations of that (st)age class, which can be proxied by considering that (st)age class' reproductive value and stable (st)age distribution. Our conclusion is generally true regardless of whether one considers continuous or discrete time, and ignorant of the exact action of genes.

## Data Availability

This article has no additional data.
